# Bridging Evidence Gaps in Geriatric Care: Large Language Models and Knowledge Graphs for Decision Support

**DOI:** 10.1093/geroni/igaf122.2243

**Published:** 2025-12-31

**Authors:** Shuqi Yang, Zheng Zhu, Shuai Wang, Jiaqing Wang, Zongan Huang, Tiantian Hu

**Affiliations:** Fudan University, Shanghai, Shanghai, China (People’s Republic); Fudan University, Shanghai, Shanghai, China (People’s Republic); Dali University, Dali, Shanghai, China (People’s Republic); Fudan University, Shanghai, Shanghai, China (People’s Republic); Fudan University, Shanghai, Shanghai, China (People’s Republic); Fudan University, Shanghai, Shanghai, China (People’s Republic)

## Abstract

The increasing prevalence of complex chronic conditions among older adults has highlighted critical gaps in the application of evidence-based practices in geriatric care. While large language models (LLMs) have demonstrated remarkable potential in medical decision support, they are prone to generating hallucinations. To address these challenges, this study developed an intelligent decision support system that integrated LLMs and knowledge graphs (KGs) to enhance evidence-based practice. The system was constructed through a multi-phase construction process: First, a high-quality geriatric symptom knowledge graph was built using structured evidence sources, including clinical guidelines and symptom networks derived from longitudinal patient data. Next, an LLM was fine-tuned with domain-specific knowledge and optimized using retrieval-augmented generation (RAG) to ensure its responses aligned with validated evidence. Finally, an interactive validation mechanism cross-referenced LLM-generated outputs with the KG, reinforcing decision accuracy and minimizing misinformation. Evaluation results demonstrated that the proposed system achieved an accuracy of 87.6%, exceeding that of a domain-specific fine-tuned LLM without KG integration (82.5%) and GPT-4 (78.3%). In terms of classification performance, the system obtained an F1 score of 85.2%, outperforming the fine-tuned LLM (80.4%) and GPT-4 (74.9%). Furthermore, the system significantly reduced the hallucination rate to 3.4% and provided rationales with citations in 92.1% of cases, ensuring enhanced transparency and reliability. These findings demonstrated that anchoring AI-generated insights in validated knowledge structures increases trust, optimizes care strategies, and supports healthcare professionals in delivering high-quality, individualized geriatric care.

